# Aerial Dissemination of *Clostridium difficile *spores

**DOI:** 10.1186/1471-2334-8-7

**Published:** 2008-01-24

**Authors:** Katherine Roberts, Caroline F Smith, Anna M Snelling, Kevin G Kerr, Kathleen R Banfield, P Andrew Sleigh, Clive B Beggs

**Affiliations:** 1 Pathogen Control Engineering Research Group, School of Civil Engineering, University of Leeds, Leeds, LS2 9JT, UK; 2 School of Engineering, Design and Technology, University of Bradford, Bradford, BD7 1DP, UK; 3 Medical Biosciences, University of Bradford, Bradford, BD7 1DP, UK; 4 Harrogate Health Care Trust, Harrogate District Hospital, Lancaster Park Road, Harrogate HG2 7SX, UK

## Abstract

**Background:**

*Clostridium difficile*-associated diarrhoea (CDAD) is a frequently occurring healthcare-associated infection, which is responsible for significant morbidity and mortality amongst elderly patients in healthcare facilities. Environmental contamination is known to play an important contributory role in the spread of CDAD and it is suspected that contamination might be occurring as a result of aerial dissemination of *C. difficile *spores. However previous studies have failed to isolate *C. difficile *from air in hospitals. In an attempt to clarify this issue we undertook a short controlled pilot study in an elderly care ward with the aim of culturing *C. difficile *from the air.

**Methods:**

In a survey undertaken during February (two days) 2006 and March (two days) 2007, air samples were collected using a portable cyclone sampler and surface samples collected using contact plates in a UK hospital. Sampling took place in a six bedded elderly care bay (Study) during February 2006 and in March 2007 both the study bay and a four bedded orthopaedic bay (Control). Particulate material from the air was collected in Ringer's solution, alcohol shocked and plated out in triplicate onto Brazier's CCEY agar without egg yolk, but supplemented with 5 mg/L of lysozyme. After incubation, the identity of isolates was confirmed by standard techniques. Ribotyping and REP-PCR fingerprinting were used to further characterise isolates.

**Results:**

On both days in February 2006, *C. difficile *was cultured from the air with 23 samples yielding the bacterium (mean counts 53 – 426 cfu/m^3 ^of air). One representative isolate from each of these was characterized further. Of the 23 isolates, 22 were ribotype 001 and were indistinguishable on REP-PCR typing. *C. difficile *was not cultured from the air or surfaces of either hospital bay during the two days in March 2007.

**Conclusion:**

This pilot study produced clear evidence of sporadic aerial dissemination of spores of a clone of *C. difficile*, a finding which may help to explain why CDAD is so persistent within hospitals and difficult to eradicate. Although preliminary, the findings reinforce concerns that current *C. difficile *control measures may be inadequate and suggest that improved ward ventilation may help to reduce the spread of CDAD in healthcare facilities.

## Background

*Clostridium difficile*-associated diarrhoea (CDAD) is a frequently occurring nosocomial infection, which is responsible for significant morbidity and mortality amongst elderly patients in healthcare facilities. In many countries the incidence of CDAD seems to be increasing and a toxin-hyperproducing strain (ribotype 027) is becoming more common [1,2], despite the fact that many hospitals have made stringent efforts to control the infection through isolation of infected patients, improved compliance with handwashing and decontamination of the ward environment. Several studies have suggested that environmental contamination, particularly of fomites, may play a role in the spread of CDAD [3-5]. Nosocomial outbreaks can occur in spatial clusters [6], suggesting that physical proximity to infected patients might be an important risk factor for acquisition of *C. difficile *[7]. This has led to suspicion that environmental contamination might be occurring as a result of aerial dissemination of *C. difficile *spores. The latter can survive on inanimate surfaces for months, and inactivating them is problematic since they are relatively resistant to disinfectants. Indeed, sporulation can actually be enhanced by exposure of cells to some types of cleaning agent [8]. Processes such as bed making are known to liberate large numbers of bacteria-carrying particles into the air [9-11] and it would appear a reasonable assumption that aerial dissemination of *C. difficile *vegetative cells and spores occurs in the same manner. However, previous studies have failed to isolate *C. difficile *from air in hospitals [3,12-14]. In an attempt to clarify this issue we undertook a short pilot study in an elderly care ward and orthopaedic ward at a 400-bedded district general hospital, with the aim of culturing *C. difficile *from the air.

## Methods

**Figure 1:**
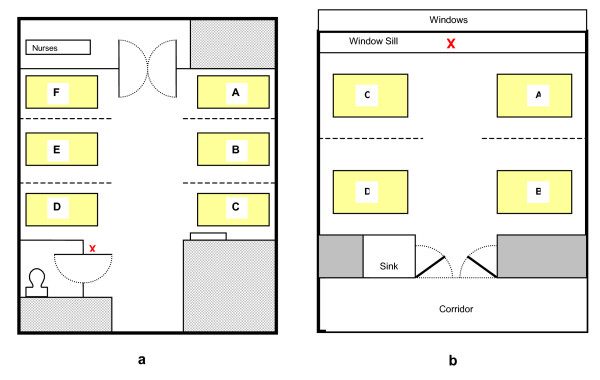
Floor plan of the study bay (a), with six open bed spaces separated by curtains (dashed lines) and the control bay (b) which contained four open bed spaces also separated by curtains. The **'X' **indicates where the air sampling equipment was positioned. T = toilet.

The first phase of the study was undertaken on the 21^st ^(day 1) and 22^nd ^February 2006 (day 2). Sampling took place in a mechanically ventilated six-bedded bay (Figure 1a) on a 29 bed elderly care ward which had sporadic cases of CDAD (four confirmed cases in 2005, and a further five confirmed cases up to March 2007). Sampling was repeated on this bay 12 months later on the 1^st ^(day 3) and 2^nd ^(day 4) of March 2007. A naturally ventilated four-bedded bay (Figure 1b) on a 15 bed orthopaedic ward, in which no CDAD cases had been reported in the previous year, was sampled concurrently during this second phase.

Ward air was sampled using a portable cyclone air sampler (Burkhard C90M, Burkhard Ltd, UK) located, in the elderly care ward, outside the entrance to the patient's toilet room (positioned on a metal trolley, 870 mm above floor level at approximately bed height) and, in the orthopaedic ward, on the window sill. During each two-day period, air samples were taken at 30-minute intervals throughout the day. For each sampling, 250 L of air was drawn into the device over 15 min. At the end of each day air samplers were cleaned with Virkon^® ^disinfectant (DuPont) which is known to be active against both *C. difficile *spores and vegetative cells. When in use, the devices were observed closely by the operator to ensure patients and staff did not touch them or interfere with their operation in any way.

Particulate matter from the air was collected into 1 mL of sterile Ringer's solution in Eppendorf tubes. Specimens were alcohol shocked [15] by mixing with an equal volume of absolute ethanol. The solution was then vortexed and held for 1 h at room temperature after which 0.1 mL aliquots were plated out in triplicate onto Brazier's CCEY agar (Lab M Ltd, Bury, UK) without egg yolk, but supplemented with 5 mg/L of lysozyme (Sigma-Aldrich, Poole, Dorset, UK) to optimise the recovery of *C. difficile *[16]. Any alcohol present was allowed to evaporate and the plates were then incubated at 37°C anaerobically for 48 h. The identity of isolates with morphology typical of *C. difficile *was confirmed using Gram staining and a commercially available latex agglutination test (Oxoid Ltd, Basingstoke, UK) which detects toxin A. Mean colony counts per cubic metre of air were calculated.

Environmental samples were taken using *Clostridium difficile *contact plates (E & O Laboratories, UK). The sites selected for testing were disinfected at the beginning of the day and after each sample was taken. In the study ward, samples were taken from the floor, top of the radiator, top of the ward door, a ventilation extract grille above bed C (see Figure 1a) and a ventilation extract grille above the nurses' station. On the control ward, samples were taken from the floor, the top of the ward door and the shelf adjacent to the hand-wash sink. Each site was screened at three set times during the day using two contact plates each time. Plates were incubated under anaerobic conditions at 37°C for 48 h.

Ribotyping [17] and repetitive extragenic palindromic REP-PCR typing [18] were used to compare the relatedness of selected isolates. *C. difficile *NCTC 11209 was used as a control for the DNA extraction and purification process. Reference strains of *C. difficile *ribotypes 001, 002, 010, 014, 027 and 106 were included as standards. Strains were cultured in Brain Heart Infusion Broth supplemented with 0.5% yeast extract and 0.5 mg of haemin, incubated anaerobically at 37°C for 24 h. Cells from 1.5 mL of culture were pelleted by centrifugation. DNA was extracted using the GenElute^TM ^Bacterial Genomic DNA kit (Sigma-Aldrich).

PCR ribotyping was performed based on a method described previously [17] using primers 5'-CTGGGGTGAAGTCGTAACAAGG-3' and 5'-GCGCCCTTTGTAGCTTGACC-3'. A 25 μl reaction mix contained 1× Thermopol II Buffer (New England Biolabs), 200 mM of each dNTP, 2.25 mM of MgSO_4_, 50 pmol of each primer (Sigma-Genosys), 2.5 units of Taq polymerase (New England Biolabs) and 2.5 μl of DNA template. The reaction mix was made up to 25 μL using molecular grade water (Sigma-Aldrich). Thirty-five cycles of amplification were carried out, consisting of 1 min at 94°C, 1 min at 55°C and 2 min at 72°C. Amplimers were resolved on a 2% agarose gel (Invitrogen). After ethidium bromide staining, banding patterns were compared visually.

REP primers REP1R-I, 5'-IIIICGICGICATCIGGC-3' and REP2-I, 5'-ICGICTTATCIGGCCTAC-3' were used in a PCR reaction based on a previously described method for typing *C. difficile *[18] with minor modifications. A 25 μL reaction mix contained 1× Thermopol II Buffer (New England Biolabs), 200 μM of each dNTP, 2.5 mM of MgSO_4_, 25pmol of each primer (Sigma-Genosys), 2 units Taq polymerase (New England Biolabs), 100 μg/mL bovine serum albumin (Promega) and DNA template (60 ng). The reaction mix was made up to 25 μL using molecular grade water (Sigma-Aldrich). Initial denaturation was 2 min at 95°C and 3 secs at 94°C. Thirty cycles of amplification were then carried out, consisting of 30 secs at 92°C, 1 min at 40°C and 2 min at 65°C. The final extension was for 15 min at 65°C. Amplimers were resolved on a 1.5% agarose gel. After ethidium bromide staining, banding patterns were compared visually.

The work described in this paper was originally undertaken as part of enhanced environmental surveillance as contained in Harrogate Health Care Trust's Infection Control Annual Plan ratified by the Trust Board. However, when it became clear that the surveillance yielded results of wider interest which might merit publication, the Trust's Research Governance Committee approved a request to submit.

## Results

**Figure 2:**
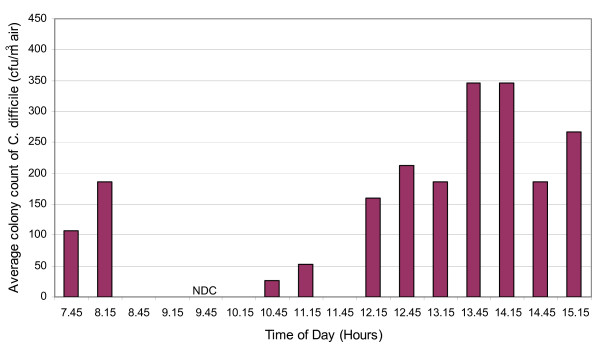
Mean *C. difficile *counts in the air of the study ward on day 1. (NB. a value of zero denotes 'below the detection limit of 27 cfu/m^3^'; NDC denotes 'no data collection').

**Figure 3:**
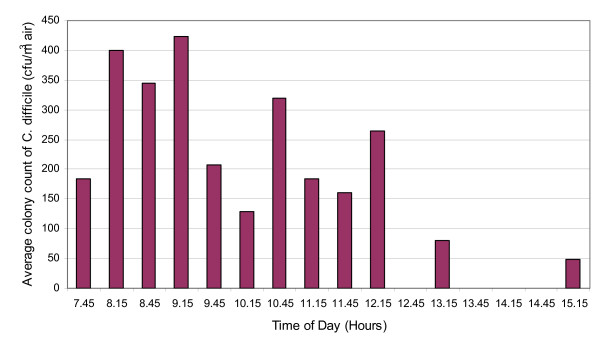
Mean *C. difficile *counts in the air of the study ward on day 2 (NB. a value of zero denotes 'below the detection limit of 27cfu/m^3^'.

The results of the first phase of air sampling are presented in Figures 2 and 3. From these it can be seen that on both days *C. difficile *was cultured from the air of the elderly care bay, with counts of 53 – 426 cfu/m^3 ^of air, indicating that substantial numbers of *C. difficile*-spores were liberated into the ward air throughout this period. The limit of detection for the method was 27 cfu/m^3 ^– this being the count that would have been recorded if a single colony was observed amongst the triplicate agar plates (1 colony arising from 0.3 ml of the alcohol shock solution = 6.67 cfu from the 250 L of air sampled, which in turn approximates to 27 per m^3^).

**Figure 4:**
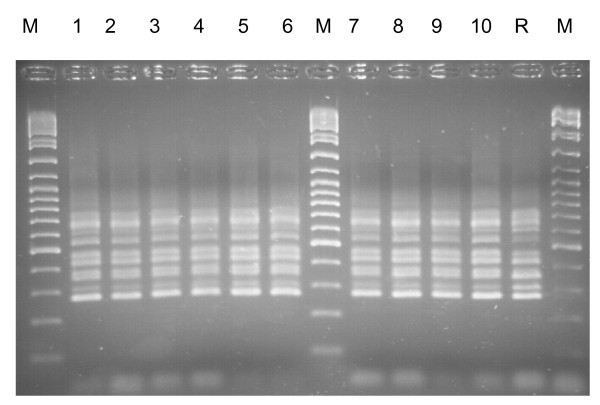
PCR ribotype profiles of *C. difficile *isolates CAS 1 to 10 (lanes 1–10) obtained from air samples, Lane R = ribotype 001 reference strain. Lane M = O'GeneRuler™ DNA ladder (10 kbp – 100 bp).

It should be noted that no sample was collected at 09.45 on day 1. Twenty-three air samples in total yielded *C. difficile *andone representative isolate (coded CAS 1 to 23) from each of these was ribotyped. Of the 23 isolates, 22 belonged to ribotype 001 (Figure 4). All of these had the same REP-PCR profile, but the pattern was different to that of the ribotype 001 reference strain (data not shown). The ribotype of the remaining isolate did not match any of the reference strains.

During the phase 1 survey, *C. difficile *was not isolated from the surfaces sampled in the elderly care ward. During the second phase of sampling, *C. difficile *was not cultured from air samples or isolated from environmental sites in both the study and control bays.

## Discussion

Earlier researchers, possibly because of sub-optimal recovery methods, such as the use of settle plates [3,12-14], failed in attempts to culture *C. difficile *from the air of hospital wards thus lending weight to the generally held opinion that airborne transmission of the bacterium is unimportant [12,19]. However, there is increasing evidence that airborne dissemination may play a role in the spread of *C. difficile *within the clinical environment. For example, air vents and high horizontal surfaces have been noted to be contaminated with *C. difficile*, [5,20] suggesting dissemination via the air. Furthermore, other studies have found *C. difficile *on patients' bedding [12-14]. As bed making is known to liberate large numbers of bacteria-carrying particles into the air [9-11], these observations would suggest that *C. difficile *may be disseminated into the air by this route following these activities. We were able to isolate *C. difficile *spores from the air on two separate days, and to our best knowledge, this is the first report to suggest aerial dissemination of this bacterium within a hospital.

The cyclone air sampler was located outside the toilet and it is possible that many of the isolates recovered from the air originated from this area. Previous work [13,14] has found surfaces within bathrooms and toilets to be among the most contaminated areas within hospitals, which is not surprising given that *C. difficile *colonises the colon. However, during the two sampling days no one was observed using the toilet as many of the patients were bed-bound. Notwithstanding this, the patient in bed D (closest to the air sampler; Figure 1) did have diarrhoea during the sampling period and used a commode on several occasions during day 2. A stool sample was negative for *C. difficile *toxin A (at tha t time the laboratory did not test for toxin B production) but this does not rule out the possibility of asymptomatic gut carriage with the bacterium [21-24]. Indeed, asymptomatic carriers are recognised as a potential cause of environmental contamination [21,22]. Before the two sampling days in February 2006, the last time a patient with confirmed CDAD was on the ward was seven weeks earlier and the next case was four weeks later. This shows that *C. difficile *can be isolated from the air in the absence of a confirmed case/outbreak of CDAD.

The results of the ribo- and REP-PCR-typing indicate that all but one of the isolates found in the air were clonal and may have come from the same source. Earlier studies have shown that some strains of *C. difficile *are more likely to contaminate the local environment than others [5,25] and the 001 ribotype cultured in this study is currently the most common strain of *C. difficile *in the UK.

Very small aerosol particles can remain airborne for long periods, for example, a 2 μm diameter particle will take 4.2 hours to fall 2 m in a still room [26]. Spores of *C. difficile *are of this size range [27] and may thus become widely distributed around the clinical environment. Aerial dissemination of desiccation-tolerant microorganisms such as *Acinetobacter *is also known to result in widespread environmental contamination [28,29].

Our data give an interesting insight into the physical nature of the *C. difficile *aerosolization and dispersal within the ward. Aerosol particles can be removed from room air by two principal mechanisms, gravitational deposition and extraction via exhaust ventilation. The residence time of true airborne particles in a well-ventilated ward space is generally ≤ 30 min, depending on the ventilation rate. Therefore, in such a space most of the airborne particles will be purged from the air within 30 min of any aerosol liberation event. From Figures 2 and 3, it can be seen that, despite some significant fluctuations, large numbers of particles were found in the air at almost all of the sampling points suggesting that numerous *C. difficile *aerosolization events occurred throughout the sampling period and also that the cyclone sampler was located close to the *C. difficile *dissemination source. It also indicates that during this period the ventilation system was unable to purge the ward air of *C. difficile*-bearing particles. Continuous observation of the device ensured that no inadvertent direct contamination (e.g. from staff/patient hands or contact with other equipment) occurred during the sampling period.

Although the study reported here was only a short pilot study, it produced evidence of aerial dissemination of *C. difficile*, a phenomenon which may, at least in part, explain why CDAD is so persistent within hospitals and difficult to eradicate. It also demonstrates the transient nature of the airborne route of dissemination, since on both sampling days in phase one there were periods where the spore count per m^3 ^of air was below the limit of detection. This has consequences for scheduling of air sampling in future studies of aerial dissemination of *C. difficile*.

It is surprising that no environmental specimens yielded *C difficile*, even on days when the bacterium was cultured from the air. This may be because we relied on 65 mm diameter contact plates. Sampling of larger areas using a moistened swab may have resulted in a greater yield of *C. difficile *[30]. Timing of sampling may also be important as sampling after ward cleaning may influence the likelihood of recovering the bacterium [30]. Furthermore, the inclusion of an agent, such as lysozyme which encourages germination of spores [16] or extending the time of incubation of cultures might have increased recovery. These issues will be addressed in future investigations.

## Conclusion

The study produced clear evidence of sporadic aerial dissemination of spores of a clone of *C. difficile*, a finding which may help to explain why CDAD is so persistent within hospitals and difficult to eradicate. As such, our report is timely because it coincides with concerns that current *C. difficile *control measures are failing to halt the spread of CDAD. Hopefully, it will encourage others to undertake aerobiological sampling in their own hospitals, so that a proper evaluation of the extent to which *C. difficile *is being disseminated via the air can be made. If airborne dissemination is a contributory factor to environmental contamination, then the use of negatively-pressurized isolation rooms and improved ward ventilation systems may help to reduce the spread of CDAD in healthcare facilities and these interventions warrant urgent evaluation.

## Competing interests

The author(s) declare that they have no competing interests.

## Authors' contributions

AMS, KGK and CBB designed the study. KR and CFS were responsible for sample collection and laboratory analysis. KRB and KGK supervised the clinical aspects of the study. KGK and AMS supervised the microbiological sampling and analysis. PAS advised on the air sampling procedures and airborne particulate behaviour. CBB wrote the manuscript with major contributions from other authors. All authors read and approved the final manuscript.

## Pre-publication history

The pre-publication history for this paper can be accessed here:


